# Fatty Acid, Lipid Classes and Phospholipid Molecular Species Composition of the Marine Clam *Meretrix lyrata* (Sowerby 1851) from Cua Lo Beach, Nghe An Province, Vietnam

**DOI:** 10.3390/molecules24050895

**Published:** 2019-03-04

**Authors:** Quoc Toan Tran, Thi Thanh Tra Le, Minh Quan Pham, Tien Lam Do, Manh Hung Vu, Duy Chinh Nguyen, Long Giang Bach, Le Minh Bui, Quoc Long Pham

**Affiliations:** 1 Institute of Natural Products Chemistry, Vietnam Academy of Science and Technology, Hanoi 122100, Vietnam; tranquoctoan2010@gmail.com (Q.T.T.); minhquanaries@gmail.com (M.Q.P.); dotienlam198@gmail.com (T.L.D.); 2 Graduate University of Science and Technology, Vietnam Academy of Science and Technology, Hanoi 122100, Vietnam; traltt@wru.vn; 3 Department of Chemical Engineering, Faculty of Environment, Thuy loi University, Hanoi 122100, Vietnam; 4 Institute of Marine Environment and Resources, Vietnam Academy of Science and Technology, Hanoi 122100, Vietnam; hungvm@imer.vast.vn; 5 NTT Hi-Tech Institute, Nguyen Tat Thanh University, Ho Chi Minh City 700000, Vietnam; ndchinh@ntt.edu.vn (D.C.N.); blgiang@ntt.edu.vn (L.G.B.); 6 Center of Excellence for Biochemistry and Natural Products, Nguyen Tat Thanh University, Ho Chi Minh City 700000, Vietnam

**Keywords:** hard clams, *Meretrix lyrata*, lipid classes, fatty acids, phospholipids, molecular species of phospholipid, high resolution mass spectrometry

## Abstract

This study aims to analyze compositions of fatty acids and phospholipid molecular species in the hard clams *Meretrix lyrata* (Sowerby, 1851) harvested from Cua Lo beach, Nghe An province, Viet Nam. Total lipid of hard clams *Meretrix lyrata* occupied 1.7 ± 0.2% of wet weight and contained six classes: hydrocarbon and wax (HW), triacylglycerol (TAG), free fatty acids (FFA), sterol (ST), polar lipid (PoL), and monoalkyl diacylglycerol (MADAG). Among the constituents, the proportion of PoL accounted was highest, at 45.7%. In contrast, the figures for MADAG were lowest, at 1.3%. Twenty-six fatty acids were identified with the ratios of USAFA/SAFA was 2. The percentage of n-3 PUFA (ω-3) and n-6 PUFA (ω-6) was high, occupying 38.4% of total FA. Among PUFAs, arachidonic acid (AA, 20:4n-6), eicosapentaenoic acid (EPA, 20:5n-3), docosapentaenoic acid (DPA, 22:5n-3), and docosahexaenoic acid (DHA, 22:6n-3) accounted for 3.8%, 7.8%, 2.2% and 12.0% of total lipid of the clam respectively. Phospholipid molecular species were identified in polar lipids of the clams consisting six types: phosphatidylethalnolamine (PE, with 28 molecular species), phosphatidylcholine (PC, with 26 molecular species), phosphatidylserine (PS, with 18 molecular species), phosphatidylinositol (PI, with 10 molecular species), phosphatidylglycerol (PG, with only one molecular species), and ceramide aminoethylphosphonate (CAEP, with 15 molecular species). This is the first time that the molecular species of sphingophospholipid were determined, in *Meretrix lyrata* in particular, and for clams in general. Phospholipid formula species of PE and PS were revealed to comprise two kinds: Alkenyl acyl glycerophosphoethanolamine and Alkenyl acyl glycerophosphoserine occupy 80.3% and 81.0% of total PE and PS species, respectively. In contrast, the percentage of diacyl glycero phosphatidylcholine was twice as high as that of PakCho in total PC, at 69.3, in comparison with 30.7%. In addition, phospholipid formula species of PI and PG comprised only diacyl glycoro phospholipids. PE 36:1 (p18:0/18:1), PC 38:6 (16:0/22:6), PS 38:1 (p18:0/20:1), PI 40:5 (20:1/20:4), PG 32:0 (16:0/16:0) and CAEP 34:2 (16:2/d18:0) were the major molecular species.

## 1. Introduction

Investigation on marine and freshwater two-part shell mollusks has been growing rapidly due to their rich nutritional value, variety of biofunctions, potent activities, and most importantly, ease of exploitation [1,2,3,4]. Poly-unsaturated fatty acids, mainly omega-3 fatty acids, have been a valuable nutrient group and the nutraceutical of interest. In almonds (*Prunus dulcis*), it is shown that the content of linoleic acid, a poly-unsaturated fatty acid, ranged from 10 to 31% depending on the origin of the sample [5]. The high content of PUFA is suggested to be associated with the cardio protective effect. The mollusk, especially bivalve mollusks, is widely consumed in Asian countries, providing high protein content, sugars, lipids, especially omega-3-fatty acids and essential amino acids, vitamin B12 and essential elements including iron, zinc and copper. Among these constituents, phospholipids and fatty acids play a major role in the functions of the immune system and the maintenance all hormonal systems of the organism. These important functionalities promote the development of new and more efficient extraction methods for mass production and commercialization of clams.

Clams, cockles, and arkshells, occupying the largest share in worldwide production of shellfish (about 6.16 million tons in 2016), are widely used as an important nutriment source [6]. Investigations of several clam species such as *Meretrix lusoria* (Asian hard clam), *Meretrix meretrix*, *Cyclina sinensis*, and *Chamelea gallina* (venus clam) have revealed that phospholipid (PL) and ω-3 long-chain polyunsaturated fatty acids (LC-PUFA) are abundantly found in clams [7,8,9,10]. Beneficial fatty acids in ω-3 LC-PUFAs include eicosapentaenoic acid (EPA, 20:5n-3), docosahexaenoic acid (DHA, 22:6n-3) and docosapentaenoic acid (DPA, 22:5n-3) [11,12,13]. Such dietary fatty acids have been studied extensively, showing a wide range of positive clinical effects including improvement in treatment of heart diseases, effective intervention of insulin sensitivity, inhibition of tumors, inflammation and metastasis. The FDA (Food and Drug Administration) has approved a qualified health claim for conventional foods and dietary supplements that contain EPA and DHA [14]. It states, “Supportive but not conclusive research shows that consumption of EPA and DHA omega-3 fatty acids may reduce the risk of coronary heart disease.” The FDA also specifies that the labels of dietary supplements should not recommend a daily intake of EPA and DHA higher than 2 g per day for health benefits [14]. For patients who need to lower their triglyceride levels, the American Heart Association recommends 2–4 g/day of EPA plus DHA under the care of a physician [15]. Several prescription omega-3 preparations are also available to treat hypertriglyceridemia [16].

Clams could be potentially a good source of PL and PUFAs. Much evidence regarding lipid composition in various clam species has been presented. For example, in the striped Venus Clam (*Chamelea gallina*), it was shown that n-3 LC-PUFA constituted a large quantity in total fatty acids of, ranging from 33.7 to 41.9%. In addition, EPA (8.2–20.0% of total fatty acids) and DHA (12.5–20.3% of total fatty acids) were also found [8]. This is also similar to the Asian hard clam (*Meretrix lusoria*), where PUFA (46.8–49.2% of total fatty acids), DHA (13.3–16.5% of total fatty acids) and EPA (4.8–7.1% of total fatty acids) were found in high levels [9]. In the *Ruditapes philippinarum* clam, PLs were the principal lipids (57–75% of total lipids) [7]. In the lipid composition of the *Calyptogena phaseoliformis* clam, major lipids are the n-4 family non-methylene interrupted polyunsaturated fatty acids (NMI-PUFA) including 20:3n-4,7,15, 20:4n-1,4,7,15, and 21:3n-4,7,16, with significant levels of 20:2n-7,15 and 21:2n-7,16 as non-methylene interrupted n-7 dienes [17]. The major fatty acids of lipids in *Meretrix lamarckii* and *Ruditapes philippinarum* were 14:0, 16:0, 18:0, 16:1n-7, 18:1n-9, 18:1n-7, 20:4n-6, 20:5n-3, and 22:6n-3, while those of *Mesolinga soliditesta* were 16:0, 18:0, 16:1n-7, 18:1n-7, 20:1n-7, 20:1n-13, 20:2n-7, 15 (Δ5,13-20:2), and 22:2n-7,15 (Δ7,15-22:2) [18].

Due to the immensity of the sea aquaculture reservoir, the Vietnamese fishery industry was ranked very highly. In terms of fish landings from marine fishing areas, Vietnam occupied the 8th position in 2015 and 2016, reaching a volume of 2.607 and 2.678 million tons respectively [19]. Among Vietnam’s highly valued aquatic organisms, the *Meretrix lyrata* (Sowerby 1851) clam, belonging to the *Veneridae* genus, also known as the hard clam, could be found on seashores and in estuarine areas. Apart from being an export product of high economic value, *Meretrix lyrata* also acts as a protein source for domestic consumption [20,21]. Considering the limited data on plasmalogens of bivalve species and the prevalence of the hard clam in Vietnamese diet, our study, for the first time, aims to report the fatty acids, lipid classes and phospholipid molecular species of the hard clams *Meretrix lyrata* S. The sample was collected in Cua Lo Beach, Nghe An Province, located in the North Central Coast region of Vietnam and populated with diversified aquaculture and ample clam species, in particular. We showed that the lipids from clams contained high percentages of PL. In addition, most of the predominant glycerophospholipid (GP) molecular species, such as phosphatidylethalnolamine (PE), phosphatidylcholine (PC), phosphatidylserine (PS), phosphatidylinositol (PI) and phosphatidylglycerol (PG) and sphingophospholipid (ceramide aminoethylphosphonate—CAEP), were identified.

## 2. Results and Discussion

### 2.1. Total Lipid

**Figure 1:**
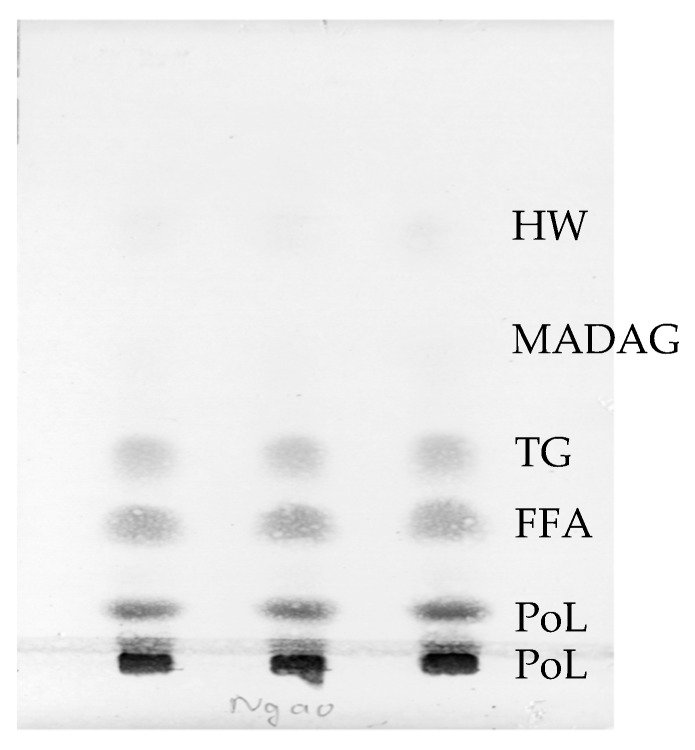
TLC determination of lipid classes of the clams.

Total lipid (TL) constituted 1.7 ± 0.2% of wet weight of the clams. The composition and content of TL of *Meretrix lyrata* (Figure 1 and Table 1) was similar to that of other clams investigated previously [18,22,23,24]. Overall, hard clam species contained common lipids hydrocarbon and wax (HW), triacylglycerol (TAG), free fatty acids (FFA), sterol (ST), polar lipid (PoL) and monoalkyl diacylglycerol (MADAG). In contrast with lipid compositions of cnidarians and coral in which MADAG accounts for a significant proportion, MADAG component of the hard clam only represents a marginal content of 1.3 ± 0.2 of TL.

The findings for PoL levels in the total lipids of the hard clam are similar to those of other studies which have investigated lipid profiles and phospholipids. In addition, this result also highlights the seasonal variation of PoL content in clam. Specifically, the analyzed hard clams, which were collected in summer, exhibited a lower PoL content in comparison to those of other clam species collected in other seasons [25,26].

Regarding other lipid classes, TAG, FFA and ST were the major lipid classes in the non-polar lipid, representing 11.7%, 18.6% and 20.2% of TL respectively. This observation was in good agreement with previous publications of other clam species [22,23,24]. Notably, the content of FFA was found to be high in hard clam, suggesting possible involvement of lipase enzymes in the intestinal organs in the hydrolysis of TAG into FFA. The presence of sterols in hard clam is also consistent with the ubiquity of the lipid class in most marine organisms, which plays many key roles, such as presenting in membrane composition [26,27].

### 2.2. Fatty Acids in Total Lipids

Twenty-six fatty acids in the lipid sample of hard clam were found with the number of carbon atoms ranging from 14 to 22 (Table 2). Major FA were Acid 16:0, 16:1n-7, 18:0, 18:1n-9, 20:4n-6 (arachidonic acid AA), 20:5n-3 (EPA) and 22:6n-3 (DHA), respectively accounting for 16.0, 6.3, 5.0, 4.7, 3.8, 7.8, and 12.0% of the total FA content. Saturated fatty acids occupied 26.3% of total FA content.

Monounsaturated fatty acids (MUFA) accounted for 15.9% of total FA content and 29.6% of unsaturated fatty acids. Two major MUFA in the composition were C16 and C18 MUFA. Regarding polyunsaturated fatty acids (PUFA), content of PUFA represented 37.7% and 70.7% of total FA content and total unsaturated fatty acids respectively. Among PUFA, three C18 PUFA were found including18:2n-6, 18:3n-3 and 18:4n-3. However, C18 PUFA only accounted for 2.9% of the total FA content. In addition, the percentages of n-6 PUFA (ω-6) were 11.2% of total FA. The figure for n-3 PUFA (ω-3) was approximately 26.5%. Highly unsaturated fatty acids (HUFA) accounted for 32.1% in total and 59.9% in unsaturated fatty acids.

These results were similar to those of various reports [23,28,29]. In the composition, several odd-chain acids were found in small quantities and methyl-branched FA including i-16:0, i-17:0, a-17:0, i-18:0 and i-21:0 were detected as trace amounts. Among many long-chain DMA produced by plasmalogen PL under acid methanolysis of clam lipids, octadecanal dimethylacetal was the major DMA and amounted to 10.8% of FA composition. Three FAs with 21 carbon atoms were identified, including 21:3n-3, 21:5n-6 and 21:5n-3 and accounted for 1.1, 1.9, and 0.7% of FA, respectively. According to a previous study [26], 6,9,12,15,18-Heneicosapentaenoic acid (21:5n-3) (HPA), was shown to exhibit stronger inhibition for arachidonic acid synthesis from alpha-linoleic acid and dihomo-gamma-linolenic acid in hepatoma cells.

### 2.3. Fatty Acids in Fractions of Phospholipid

As shown in Table 3, GC analysis indicated that the fractions of phospholipid of the clams contained a high proportion of SAFAs. Saturated fatty acids exist dominantly in CAEP, at 75.8%. This figure was more than twice as much as of PS + PI. The figures for PE + PG and PC were lower, at 40.5, and 52.2% respectively. The abundance of SAFAs in the FA composition of CAEP was consistent with the molecular species content of 16:0, 17:0, and 18:0 acid, which were reported later in this study and totaled at 67.1% of CAEP from hard clam (Table S6).

Compared with the lipids recovered from the other clam species [17], the FA composition results of hard clam were also similar regarding DMA in PE and PC fraction. To be specific, both studies suggested that DMAs were non-existent in FA composition of PC in investigated clam species. In addition, present results also showed that CAEP fraction contains no DMA and PE is the only fraction which contains all three DMAs including of 16:0, 17:0 and 18:0 DMA, with corresponding contents of 0.4, 1.2, and 11.3%. In fractions of PS + PI, only 17:0 DMA and 18:0 DMA exists at 1.5% and 14.6% respectively. Thus, two kinds of phospholipid formula species contained in PE and PS were plasmenyl and diacyl phospholipids and two phospholipid formula species of PC includes plasmanyl and diacyl glycero phospholipids.

### 2.4. Molecular Species of Phospholipid

Molecular species of polar lipids from the hard clam were determined following the previously described HRMS fragmentations of PL standards [30]. TL of Clams contained 48.96% of polar lipids (PoL) (Table 1). PoL have got glycolipid and phospholipid. Especially in the hard clams, phospholipid dominated, with six types of phospholipids, including phosphatidylethanolamine (PE), phosphatidylcholine (PC), phosphatidylserine (PS), phosphatidylinositol (PI) and phosphatidylglycerol (PG) and Ceramide aminoethylphosphonate (CAEP). The molecular species and qualification of each phospholipid were performed by HPLC-HRMS [31].

Among phosphatidylethanolamine (PE) of clams, we determined 28 constituents. Alkenyl acyl glycerophosphoethanolamine (ethanolamine plasmalogen PlsEtn) was the major component in PE, with 80.3% of total PE species (Table S1), followed by PE 40:6 (*m*/*z* (M+) 776.5556), at 17.13%, Plasmalogen PE 36:1 (*m*/*z* (M+) 730.5794), at 15.4%, and PE 40:2 (*m*/*z* (M+) 784.6161), at 10.3%.

**Figure 2:**
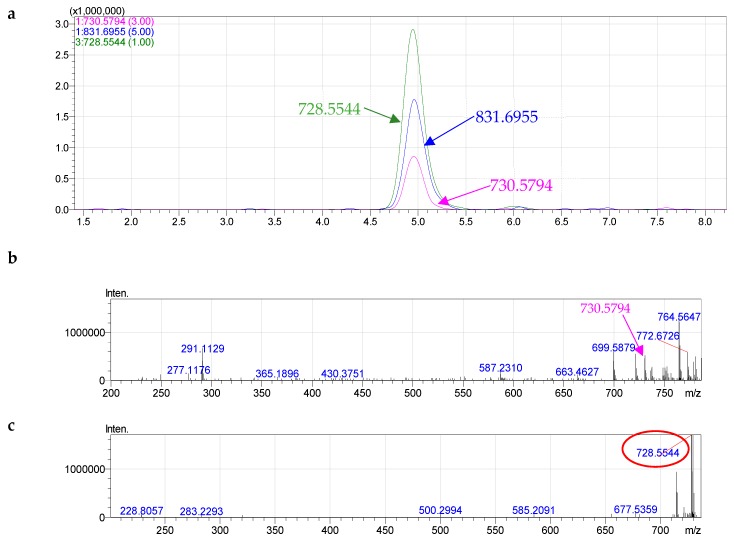

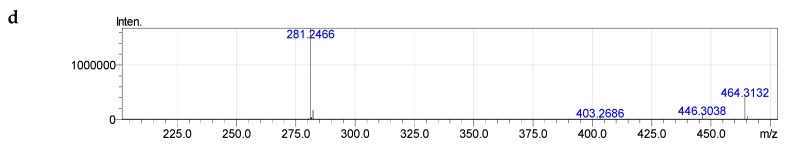
HPLC–HRMS (**a**) and fragmentation of PE 36:1(**b**—MS^+^, **c**—MS^−^, **d**—MS^2−^).

PlsEtn can be characterized according to their MS and MS/MS data. The signals of positive quasi-molecular ions [M + H]^+^, cluster ions [M + H + C_6_H_15_N]^+^ and negative quasi-molecular ions [M − H]^−^ were observed in the HRMS spectra of all components of formula species of PE. For instance, we detected signals of negative quasi-molecular ions [M − H]^−^ at *m*/*z* 728.5544 ([C_41_H_79_NO_7_P]^−^, calculated 728.5594, different 0.00556), positive quasi-molecular ions [M + H]^+^ at *m*/*z* 730.5794 ([C_41_H_81_NO_7_P]^+^, calculated 730.5751, different 0.00488), and positive cluster ions [M + C_6_H_15_N]^+^ at *m*/*z* 831.6822 ([C_47_H_96_N_2_O_7_P]^+^, calculated 831.6955, different 0.01277) of PE 36:1 (Figure 2).

From the the MS^2−^ spectrum of the ions [M − H]^−^ of PE 36:1, one signal corresponding to one carboxylate anion of 18:1 was detected at *m*/*z* 281.2466. For the plasmalogen, the fatty acid (FA) anion ([RCOO]^−^) can only be liberated from the *sn*-2 position due to the ankenyl linkages at the *sn*-1 position [32]. Therefore, three possible molecular species including plasmenyl p18:0/18:1. Component PE 36:1 was identified as alkenyl acyl glycerophosphoethanolamine, p18:0/18:1 PlsEtn (Figure S1).

Diacyl glycerophosphoethanolamine only accounted for 19.7% of total PE species. For an unknown PL containing two FAs, MS and MS/MS data also are needed to determine the two individual FAs esterified at the *sn*-1 and *sn*-2 positions of the glycerol backbone.

**Figure 3:**
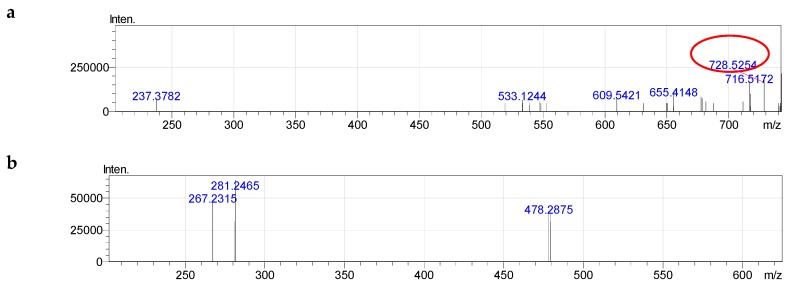
Fragmentation of PE (35:2): **a**—MS^−^, **b**—MS^2−^.

For example, for the unknown PE with measured *m*/*z* of 730.5411 (quasi-molecular ion ([M + H]^+^)), PE 35:2 could tentatively be deduced according to the previously described formulae [30,31]. The MS^2−^ spectrum of the ions [M − H]^−^ of component PE 35:2 contained the signal of two carboxylate anion of 18:1 at *m*/*z* 281.2466 and 17:1 at *m*/*z* 267.2315 (Figure 3 and Figure S2). The component PE 35:2 was determined as diacy glycerophosphoethanolamine, 18:1/17:1.

The choline glycerophospholipids (ChoGpl) of clams is summarized in Table S2. Among 26 components, 9 of which constituted more than 51% of total ChoGpl. The percentage of diacyl glycero phosphatidylcholine was twice as abundant as PakCho in total PC, at 69.3% in comparison with 30.7%.

**Figure 4:**
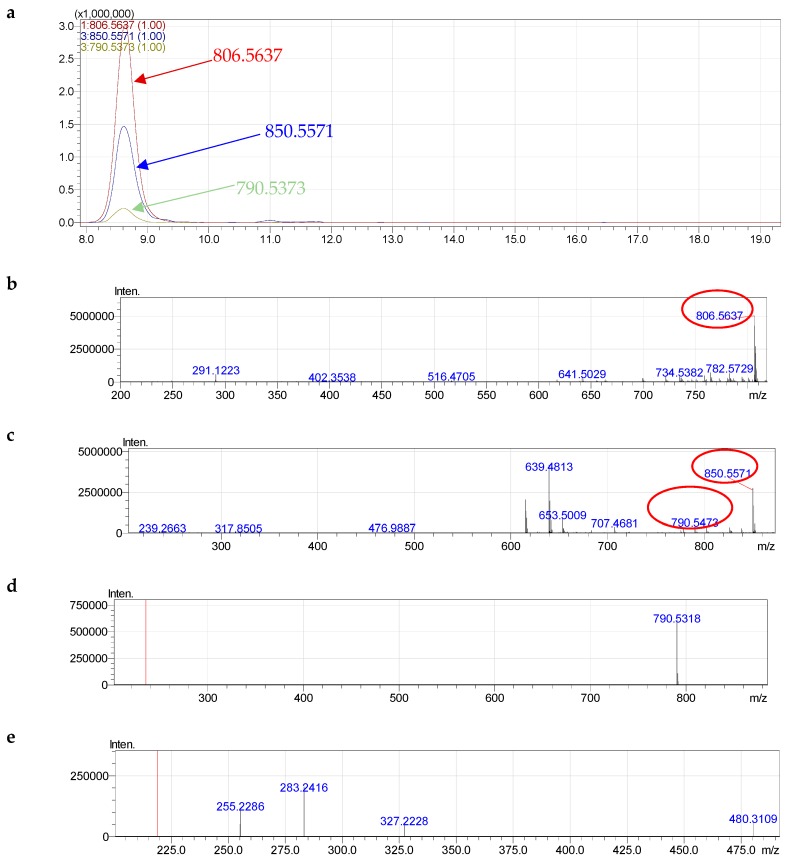
Fragmentation of PC (38:6): **a**—HPLC-HRMS, **b**—MS^+^; **c**—MS^−^ of fragmentation 850.5490 and 790.5301; **d**, **e**—MS^2−^ of fragmentation 850.5490 and 790.5301.

The signals of positive quasi-molecular ions [M + H]^+^, negative formate molecular ions [M + HCOO]^−^ and cluster ions [M − CH_3_]^−^ were observed in the HRMS spectra of all components of formula species of PC. Formate molecular ions ([M + HCOO]^−^) of each component lost methyl formate at the MS^2−^ stage (Figure 4). For instance, with the highest percentage at 16.0%, PC 38:6 formed negative acetylated molecular ions [M + HCOO]^−^ at *m*/*z* 850.5571, positive quasi-molecular ions [M + H]^+^ at *m*/*z* 806.5637 and cluster ions [M − CH_3_]^−^ at 790.5373 corresponding to composition [C_47_H_81_NO_10_P]^−^ (calculated850.5598, different 0.00326), [C_46_H_81_NO_8_P]^+^ (calculated806.5700, different 0.00573) and [C_45_H_77_NO_8_P]^−^ (calculated 790.5387, different 0.00193), respectively (Figure 4).

At the MS^2−^ stage, the ions at *m*/*z* 850.5571 eliminated a molecule of C_2_H_4_O_2_ (methyl formate) and formed ions at *m*/*z* 790.5303, suggesting the formation of the quasi-molecular negative ions by the addition of formate ion to the lipid molecule. On MS^2−^ of component lost methyl formate, two carboxylate anion of 22:6 at *m*/*z* 327.2228 (calculated 327.2324, different 0.0105) and 16:0 at *m*/*z* 255.2286 (calculated 255.2324, different 0.00435) were observed (Figure 4 and Table S3). Normally, the *sn*-2 of PL is the preferred position for PUFAs [32]. Therefore, PC 38:6 was characterized as diacy glycerophosphocholine 16:0/22:6.

Among phosphotidylserine PS of clams, we determined 18 constituent components (Table S3). Alkenyl acyl glycerophosphoserine was the major component in PS, with 11 components occupying 81.0% of total PS species.

**Figure 5:**
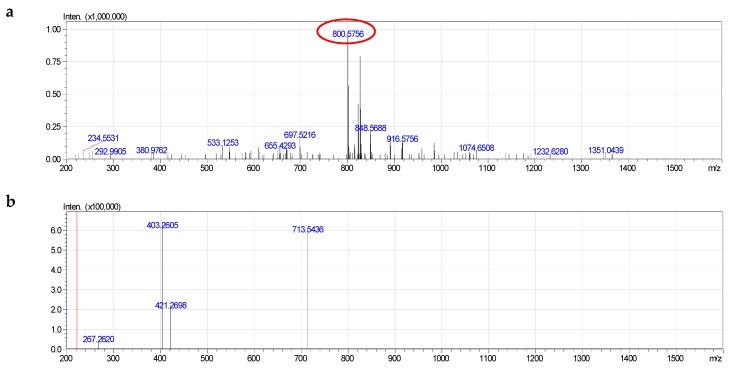
Fragmentation of PS (38:1): **a**—MS^−^, **b**—MS^2−^.

All components of formula species of PS had a signal of negative quasi-molecular ions [M − H]^−^. On the other hand, positive quasi-molecular ions did not form. The MS^2−^ spectrum of [M − H]^−^ of each component contained a signal of characteristic ion [M–H–C_3_H_5_NO_2_]^−^ corresponding to the loss of serine group (Figure 5). This was a specific fragmentation difference from the fragmentations of negative quasi-molecular ions of PE.

For example, PS 38:1 formed negative quasi-molecular ion [M − H]^−^ at 800.5756 ([C_44_H_83_NO_9_P]^−^, calculated 800.5805, different 0.00549). On MS^2−^, the absence of serine (713.6436) and serine and acyl groups (403.2605) was observed. However, signal of fatty acid was not detected (Figure S4). The component PS 38:1 was determined as alkenyl acyl glycerophosphoserine, p18:0/20:1.

**Figure 6:**
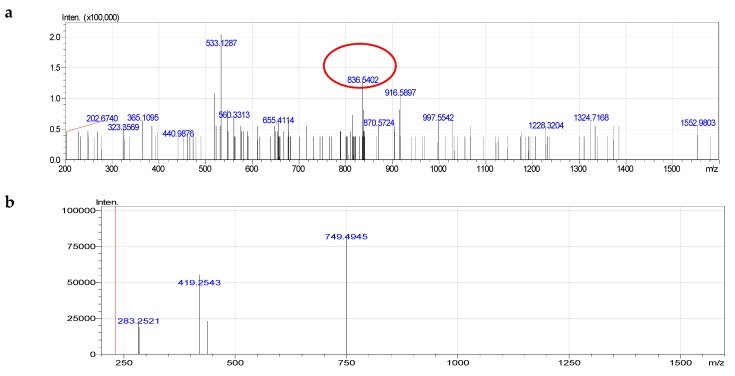
Fragmentation of PS (40:5): **a**—MS^−^, **b**—MS^2−^.

Diacyl glycerophosphoserines only accounted for 19% of total PS species. On the MS^2−^ of signal negative quasi-molecular ion [M-H]^−^ at 836.5402 ([C46H79NO10P]-, mass 836.5402, different 0.00451), the signal lost serine (749.4945) and lost simultaneously serine and acyl groups (419.2543) and one carboxylate anion of fatty acid 18:0 at *m*/*z* 283.2521 (Figure 6 and Figure S5). The component PS 40:5 was determined as diacy glycerophosphoserine, 18:0/22:5.

Among phosphotidylinositol PI of clams, we determined 10 components (Table S4). In addition, Alkenyl acyl glycerophosphoinositol was absent in PI. All ten components PI were diacyl glycerophosphoinositol with fatty acids 16:0. 17:0, 18:0, 19:0, 20:1, 20:4, 20:5.

**Figure 7:**
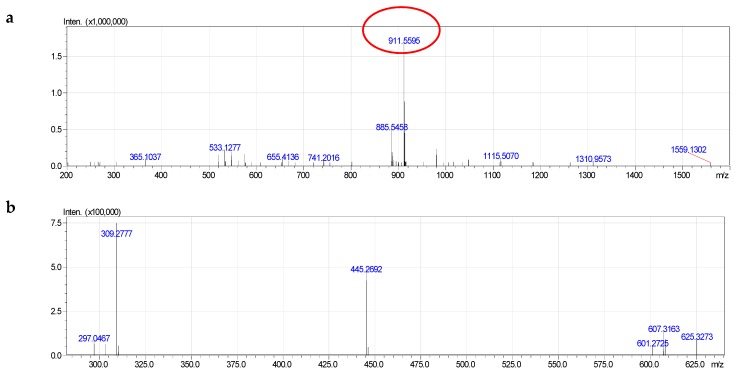
Fragmentation of PI (40:5): **a**—MS^−^, **b**—MS^2−^.

The molecular species of clam PtdIns indicated the formation of both negative and positive quasi-molecular ions [M − H]^−^. In addition, MS^2−^ fragmentation of the ions [M − H]^−^ of PtdIns also reveals the presence of several characteristic ions such as PI 40:5 (Figure 7 and Figure S6). The fragmentation 911.5593 had formula [C_49_H_84_O_13_P]^−^, calculated 911.5650 (different 0.00620). The MS^2−^ spectra of component PI 40:5 also contained signals corresponding to the quasi-molecular ion, whose inositol, acyl groups and carboxylate anion of fatty acid have been simultaneously lost. In addition, fragmentation 297.0467 was quasi-molecular ion that lost diacyl groups. This fragmentation was important in determining the molecular species of PI.

The component PI 40:5 (Figure S6) was determined as diacy glycerophosphoinositol 20:1/20:4.

We determined a single component, which was isomers of PG 32:0 constituting phosphatidylglycerol (PG) (Table S5).

**Figure 8:**
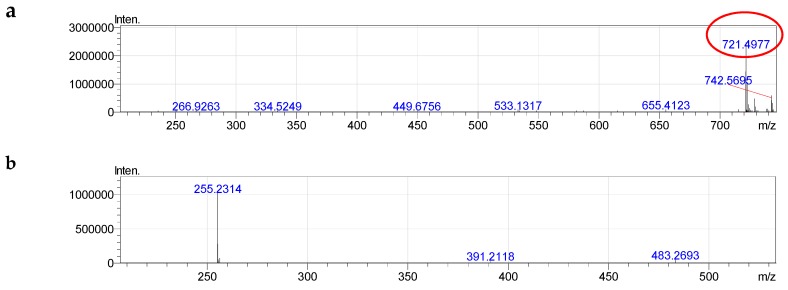
Fragmentation of PG (32:0): **a**—MS^−^, **b**—MS^2−^.

PG 32:0 was described as follows (Figure 8 and Figure S7). The MS^2−^ spectra of component PG 32:0 contained signals corresponding to the negative quasi-molecular ion 721.4975 ([C_38_H_74_O_10_P]^−^, calculated 721.5020, different 0.00501). On MS^2−^, we obtained fragmentations of carboxylate anion of fatty acid 16:0 (255.2304) with the simultaneous absence of glycerol and acyl groups (391.2255).

The component PG 32:0 (Figure S7) was determined as diacy glycerophosphoglycerol 16:0/16:0.

Fifteen molecular species of CAEP were determined (Table S6). In mass spectra of all molecular species, we observed signals of negative quasi-molecular ions [M − H]^−^, positive quasi-molecular ions [M + H]^+^ and positive quasi-molecular ions [M + Na]^+^.

**Figure 9:**
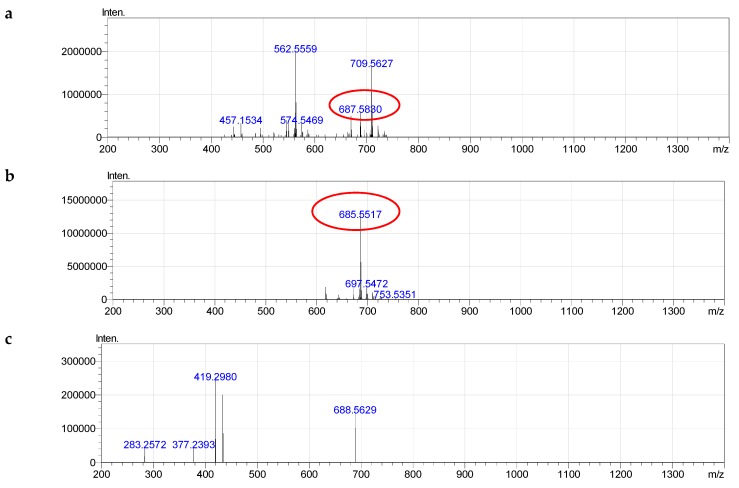
Fragmentation of CAEP (37:1): **a**—MS^+^, **b**—MS^−^, **c**—MS^2−^.

For example, we had signals of negative quasi-molecular ions [M − H]^−^, at *m*/*z* 685.5565 ([C_39_H_78_N_2_O_5_P]^−^, calculated 685.5648, different 0.00888), positive quasi-molecular ions [M + H]^+^ at *m*/*z* 687.5830 ([C_39_H_80_N_2_O_5_P]^+^, calculated 687.5805, different 0.00306), and positive adductions [M + Na]^+^ at *m*/*z* 709.5627 ([C_39_H_79_N_2_O_5_NaP]^+^, calculated 709.5619, different 0.00082) of CAEP 37:1 (Figure 9).

The MS^2−^ spectrum of [M − H]^−^ of CAEP which was sphingolipid signified a specific fragmentation similar to the fragmentations of negative quasi-molecular ions of glycerophospholipids (Figure S8). On MS^2−^, we obtained carboxylate anion fragmentation of fatty acid 18:0 (283.2572). In addition, [M − H]^−^ lost a neutral fragment amounted to 266.2567 and formed a single ion at 419.2980. Other fragmentations are also explained in Figure S8.

## 3. Materials and Methods

### 3.1. Material

The colonies of the clams were collected in May 2017 in Cua Lo beach, Cua Lo town, Nghe An provine, Vietnam.

The samples were transported immediately to the Institute of Natural Products Chemistry, Vietnam Academy of Science and Technology. Then, the shells and the meat were separated and stored at −5 °C.

### 3.2. Total Lipid Extraction

Soft tissue of clams was crushed and total lipid (TL) was extracted using modified Blight–Dyer extraction technique [31]. To be specific, lipids were extracted by homogenizing with the CHCl_3_/CH_3_OH solution (1:2, *v*/*v*) (30 mL per 10 g of clams wet weight) in 6 h, at 30 °C. The obtained homogenate was filtered, and the residue was repeatedly extracted (6 h, 30 °C) in CHCl_3_ (20 mL) a second time. After extraction, the homogenates were then mixed and separated into layers by adding 20 mL of H_2_O. The lower layer was evaporated and the obtained TL obtained was dissolved in CHCl_3_ and stored at −5 °C.

### 3.3. Analyses of Lipid Classes

To determine lipid class compositions, the extracted lipids were first dissolved in chloroform (10 mg/mL) and then spotted onto the one-dimensional thin-layer chromatography (TLC) using the pre-coated silica gel plates (6 cm × 6 cm) Sorbfil PTLC-AF-V (Sorbfil, Krasnodar, Russia). The first development of the plate was carried out with n-hexane/diethyl ether/acetic acid (85:15:1, *v*/*v*/*v*) for full length. Then, the plate was redeveloped with CHCl_3_/CH_3_OH (2:1, *v*/*v*) for 5% length. Subsequently, the plates were air-dried, sprayed with 10% H_2_SO_4_/CH_3_OH and heated at 240 °C for 10 min. An image scanner (Epson Perfection 2400 Photo, Epson, Suwa, Japan) operating in grayscale mode was employed to obtain chromatograms. For determination of lipid percentages, band intensity was evaluated using an image analysis program (Sorbfil TLC Video densitometer, Krasnodar, Russia).

### 3.4. Separation of Phospholipid Classes

The TLC plates used in this study were 10 × 10 cm glass-backed HPTLC Silica gel 60 plates (Merck, Darmstadt, Germany). Prior to sample loading, the plates were activated at 110 °C for at least 90 min and allowed to cool to room temperature in a vacuum desiccator. One dimensional TLC was employed to analyze phospholipids with the solvent system of chloroform–methanol–25% aqueous ammonia–benzene (65:30:6:10, *v*/*v*/*v*/*v*) [33]. Phosphomolybdate spray reagent for detection was prepared following a previous study [34].

Spots from TLC plates was eluted by the following procedure. First, for each phospholipid, corresponding band was scraped from the plate developed twice in 1st direction with the aforementioned solvent system. Second, elution was performed with chloroform–methanol (1:1, *v*/*v*).

### 3.5. Analyses of Fatty Acids

To obtain fatty acid methyl esters (FAME), the lipids were treated with 2% H_2_SO_4_ in CH_3_OH in a screw-capped vial (2 h, 80 °C) under air and purified by TLC development in hexane–diethyl ether (95:5, *v*/*v*). FAME were analyzed with GC at 210 °C and identified with reference to authentic standards and a table of equivalent chain lengths [35]. Injector and detector temperatures were 240 °C.

To confirm structures of FA, corresponding FAME were analyzed with GC–MS and resulted spectra were matched with the NIST library and FA mass spectra archive [36]. Temperature of the GC–MS analysis initiated at 160 °C and then progressed at 2 °C/min to 240 °C which was kept constant for 20 min. Injector and detector temperatures were 250 °C.

### 3.6. Analysis of Molecular Species of Phospholipids

Phospholipids were analyzed with high performance liquid chromatography–high resolution mass spectrometry (HPLC–HRMS) to identify and quantify chemical structures of molecular species of phospholipids.

The high performance liquid chromatography (HPLC) separation of PL was performed at the constant content of (C_2_H_5_)_3_N/acid formic (0.08:1, *v*/*v*) in the solvent system [37] that allowed carrying out efficient ionization in ESI conditions and obtaining a stable ion signal by the simultaneous registration of positive and negative ions. For polar lipids, HPLC separation was performed using the binary solvent gradient consisted of solvent mixture A: *n*-hexane/2-propanol/acid formic/(C_2_H_5_)_3_N (82:17:1:0.08, *v*/*v*/*v*/*v*) and mixture B: 2-propanol/H_2_O/acid formic/(C_2_H_5_)_3_N (85:14:1:0.08, *v*/*v*/*v*/*v*). The gradient started at 5% of mixture B, and its percentage was increased to 80% over 25 min. This composition was maintained for 1 min before being returned to 5% of mixture B over 10 min and maintained at 5% for another 4 min (the total run time was 40 min). The flow rate was 0.2 mL/min. Polar lipids were detected by high resolution mass spectrometry (HRMS) and identified by a comparison with authentic standards using a Shimadzu LCMS Solution control and processing software (v.3.60.361, Shimadzu, Kyoto, Japan). The quantification of individual molecular species within each polar lipid class was carried out by calculating the peak areas for the individual extracted ion chromatograms [38].

### 3.7. Instrumental Equipment

The GC analysis was carried out on a Shimadzu GC-2010 chromatography (Kyoto, Japan) with a flame ionization detector on a SUPELCOWAX 10 (Supelco, Bellefonte, PA, USA) capillary column (30 m × 0.25 mm × 0.25 μm). Carrier gas was He at 30 cm/s. The GC–MS analysis was performed with a Shimadzu CMS-QP5050A instrument (Kyoto, Japan) (electron impact at 70 eV) with a MDN-5s (Supelco, Bellefonte, PA, USA) capillary column (30 m × 0.25 mm ID). Carrier gas was He at 30 cm/s.

The HPLC–HRMS analysis of polar lipids was performed with a Shimadzu Prominence liquid chromatograph equipped with two LC-20AD pump units, a high pressure gradient forming module, CTO-20A column oven, SIL-20A auto sampler, CBM-20A communications bus module, DGU-20A3 degasser, and a Shim-Pack diol column (50 mm × 4.6 mm ID, 5 μm particle size) (Shimadzu, Kyoto, Japan). Lipids were detected by a high resolution tandem ion trap–time of flight mass spectrometry with a Shimadzu LCMS-IT-TOF instrument (Kyoto, Japan) operating both at positive and negative ion mode during each analysis at electrospray ionization (ESI) conditions. Ion source temperature was 200 °C, the range of detection was m/z 200–1600, and potential in the ion source was −3.5 and 4.5 kV for negative and positive modes, respectively. The drying gas (N_2_) pressure was 200 kPa. The nebulizer gas (N_2_) flow was 1.5 L/min.

## 4. Conclusions

Lipid analysis of the present study revealed that the total lipid in hard clam (*Meretrix lyrata*) occupies 1.7 ± 0.2% of wet weight and that six lipid classes consisting of hydrocarbon and wax (HW), triacylglycerol (TAG), free fatty acids (FFA), sterol (ST), polar lipid (PoL) and monoalkyl diacylglycerol (MADAG) were detected. Among these classes, the proportion of PoL and MADAG accounted for the highest, at 45.7%, and the lowest, at 1.3% of total lipid, respectively. In addition, the ratios of PUFA/SAFA was 1.4. The total percentage of n-3 PUFA (ω-3) and n-6 PUFA (ω-6) was relatively high, at 38.4% of total FA.

To our knowledge, this is the first report to determine chemical structures and quantities of molecular species of phospholipid, in general, and phosphoethanolamines in particular, on the hard clams *Meretrix lyrata* from Cua Lo beach, Nghe An province, Viet Nam. Five types glycerophospholipid were identified: PE, PC, PS, PI and PG. One type sphingophospholipid was identified to be CAEP. Ninety-eight molecular species were identified in polar lipids of the clams. Alkenyl acyl forms of glycerophospholipids predominated in the molecular species determined. PE 36:1 (p18:0/18:1), PC 38:6 (16:0/22:6), PS 38:1 (p18:0/20:1), PI 40:5 (20:1/20:4), PG 32:0 (16:0/16:0) and CAEP 34:2 (16:2/d18:0) were the major molecular species.

## Figures and Tables

**Table 1:** Composition of main lipid classes (% of total lipid) of the clams.

No.	Lipid Class	Content (%)
1	Hydrocarbon and wax (HW)	2.6 ± 0.1
2	Monoalkyl diacylglycerol (MADAG)	1.3 ± 0.2
3	Triacylglycerol (TG)	11.7 ± 0.3
4	Free fatty acids (FFA)	18.6 ± 0.8
5	Sterols (ST)	20.2 ± 0.5
6	Polar lipid (PoL)	45.7 ± 0.6

**Table 2:** Fatty acid composition (% of total) of clams.

	R.t (min)	Area	Content (%)
Σ SAFAs		1,971,006	26.3
14:0 *	3.748	291,352.4	3.9
16:0 *	5.491	1,197,827	16.0
17:0 *	6.812	105,581.2	1.4
18:0 *	8.686	376,245.5	5.0
Σ MUFAs		1,191,078	15.9
16:1n-7 *	5.863	469,554.2	6.3
18:1n-9 *	9.228	355,129.6	4.7
18:1n-7 *	9.378	147,985.6	2.0
20:1n-11	15.324	99,722.7	1.3
20:1n-9 *	15.511	42,995.4	0.6
20:1n-7 *	15.875	75,690.3	1.0
Σ PUFAs		2,831,606	37.7
16:3n-3	7.292	56,506.2	0.8
18:2n-6 *	10.411	63,571.7	0.8
18:3n-3	12.383	77,148.8	1.0
18:4n-3 *	13.518	80,259.3	1.1
20:2n-6 *	17.775	108,771.3	1.4
20:3n-6	19.233	31,176.5	0.4
20:4n-6 *	20.617	283,411	3.8
20:4n-3 *	23.19	36,235.8	0.5
20:5n-3 *	24.929	588,733	7.8
21:3n-3	28.661	85,752.1	1.1
21:5n-6	29.156	144,854.4	1.9
21:5n-3	33.552	53,585.8	0.7
22:4n-6 *	36.426	115,438.2	1.5
22:5n-6	39.584	96,337.1	1.3
22:5n-3 *	44.249	161,865.8	2.2
22:6n-3 *	48.476	901,545.1	12.0
Octadecanal ^a^	7.882	808,630.9	10.8
Other ^b^		702,316.4	9.4

* Fatty acids were identified by GCMS and GC. ^a^ Fatty aldehyde was determined as its dimethylacetal derivatives by GC and GCMS. ^b^ 15:0, i-16:0, 16:1n-5, i-17:0, a-17:0, i-18:0, 17:3n-6, a-19:0, 19:0, 18:3n-6, 19:1n-9, 19:1n-7, 20:0, i-21:0, a-21:0, 20:3n-3, hexadecanal, hexadecenal, octedecenal. SAFAs: saturated fatty acids. MUFAs: monounsaturated fatty acids. PUFAs: poliunsaturated fatty acids.

**Table 3:** Fatty acid composition (% of each type phospholipid) of clams.

Fraction	PE + PG	PC	CAEP	PS + PI
Σ SAFAs	40.5	52.2	75.8	32.5
14:0	4.3	nd	nd	0.7
16:0	22.0	36.9	57.0	12.9
17:0	3.2	4.1	4.3	2.1
18:0	11.0	9.9	13.0	16.2
20:00	nd	1.3	1.5	0.4
Σ MUFAs	25.3	14.4	11.3	22.5
16:1n-7	10.9	3.6	nd	1.2
18:1n-9	8.4	7.9	8.5	9.5
18:1n-7	3.5	1.5	0.4	0.7
20:1n-11	1.1	0.5	0.3	7.4
20:1n-9	0.6	0.5	0.7	1.2
20:1n-7	0.8	0.4	1.4	2.5
Σ PUFAs	14.9	29.9	9.1	22.7
16:3n-3	0.9	0.8	nd	nd
18:2n-6	0.9	1.0	2.1	1.5
18:3n-3	1.2	0.5	0.1	nd
18:4n-3	0.8	0.6	nd	nd
20:2n-6	nd	1.2	0.2	nd
20:3n-6	nd	0.7	0.3	nd
20:4n-6	1.7	2.4	1.7	5.8
20:4n-3	nd	0.3	nd	nd
20:5n-3	2.9	3.8	1.2	1.4
21:3n-6	nd	2.6	0.8	nd
21:3n-3	2.2	0.4	0.2	4.9
21:5n-6	nd	0.8	0.3	nd
21:5n-3	nd	0.1	1.1	nd
22:4n-6	nd	1.0	nd	0.7
22:5n-6	1.1	0.9	nd	1.5
22:5n-3	nd	1.8	nd	1.3
22:6n-3	3.2	11.0	1.0	5.5
Σ DMAs	12.9			16.0
16:0	0.4	nd	nd	nd
17:0	1.2	nd	nd	1.5
18:0	11.3	nd	nd	14.6
Other	6.3	3.5	3.8	6.3

nd: not detected or under 0.1% of fatty acid composition in each type phospholipid. SAFAs: saturated fatty acids. MUFAs: monounsaturated fatty acids. PUFAs: poliunsaturated fatty acids. DMAs: dimethylaxetal. PE: Phosphatidylethanolamine. PC: Phosphatidylcholine. PS: Phosphatidylserine. PI: Phosphatidylinositol. PG: Phosphatidylglycerol. CAEP Ceramide aminoethylphosphonate.

## Supplementary Materials

The supplementary materials are available online.

## Abbreviations

AA	Arachidonic acid
CAEP	Ceramide aminoethylphosphonate
ChoGpl	Choline glycerophospholipids
DHA	Docosahexaenoic acid
DMA	Dimethylacetal
DPA	Docosapentaenoic acid
EPA	Eicosapentaenoic acid
FFA	Free fatty acids
GC	Gas chromatography
GCMS	Gas chromatography—mass spectrometry
HPA	Heneicosapentaenoic acid
HPLC-HRMS	High performance liquid chromatography–high resolution mass spectrometry
HRMS	High resolution mass spectrometry
HUFA	Highly unsaturated fatty acid
HW	Hydrocarbon and wax
LC-PUFA	Long-chain polyunsaturated fatty acid
MADAG	Monoalkyl diacylglycerol
MS	Mass spectrometry
MS/MS	Mass spectrometry / Mass spectrometry (or tandem mass spectrometry)
MUFA	Monounsaturated fatty acid
NMI-PUFA	Non-methylene interrupted polyunsaturated fatty acid
PakCho	1-*O*-alkyl-2-acyl-*sn*-glycero-3-phosphocholine
PC	Phosphatidylcholine
PE	Phosphatidylethalnolamine
PG	Phosphatidylglycerol
PI	Phosphatidylinositol
PL	Phospholipid
PlsEtn	Ethanolamine plasmalogen
PoL	Polar lipid
PS	Phosphatidylserine
PtdIns	Phosphatidylinositol
PUFA	Poly-unsaturated fatty acid
SAFA	Saturated fatty acid
ST	Sterol
TAG	Triacylglycerol
TL	Total lipid
TLC	Thin-layer chromatography
USAFA	Unsaturated fatty acid
