# Supplementary material for: Fatty Acid, Lipid Classes and Phospholipid Molecular Species Composition of the Marine Clam Meretrix lyrata (Sowerby 1851) from Cua Lo Beach, Nghe An Province, Vietnam
Source: Molecules. 2019 Mar 4;24(5):895. doi: 10.3390/molecules24050895 (PMC6429517; doi:10.3390/molecules24050895)
Supplement: Supplementary file 1 [file molecules-24-00895-s001.pdf]

Supplementary A: Tables

Table S1. The composition of molecular species of phosphatidylethalnolamine (PE) from the clams.

PL	Symbol	M+	Formula	Area	% in PE
PE 33:1	p17:0/16:1	688.5269	$C_{38}H_{74}NO_7P$	1497699	1.6
PE 34:1	p18:0/16:1 p16:0/18:1	702.5410	$C_{39}H_{76}NO_7P$	5712376	6.0
PE 35:1	p17:0/18:1	716.5575	$C_{40}H_{78}NO_7P$	4716264	5.0
PE 36:1	p18:0/18:1	730.5794	$C_{41}H_{80}NO_7P$	14652116	15.4
PE 38:5	p18:0/20:5	750.5444	$C_{43}H_{76}NO_7P$	3211714	3.4
PE 38:2	p18:0/20:2	756.5835	$C_{43}H_{82}NO_7P$	5720469	6.0
PE 38:1	p18:0/20:1	758.6006	$C_{43}H_{84}NO_7P$	1178033	1.2
PE 39:7	p17:0/22:6	762.5406	$C_{44}H_{76}NO_7P$	1574842	1.7
PE 39:5	p17:0/22:5	764.5537	$C_{44}H_{78}NO_7P$	2309139	2.4
PE 39:1	p17:0/22:2	770.6018	$C_{44}H_{84}NO_7P$	1566703	1.6
PE 40:6	p18:0/22:6	776.5556	$C_{45}H_{78}NO_7P$	16448856	17.3
PE 40:5	p18:0/22:5	778.5704	$C_{45}H_{80}NO_7P$	4784699	5.0
PE 40:3	p18:0/22:3	782.5966	$C_{45}H_{84}NO_7P$	2470761	2.6
PE 40:2	p18:0/22:2	784.6161	$C_{45}H_{86}NO_7P$	9756482	10.3
PE 41:2	p19:0/22:2	798.6334	$C_{46}H_{88}NO_7P$	741412	0.8
PE 34:2	16:1/18:1	716.5323	$C_{39}H_{74}NO_8P$	646687	0.7
PE 34:1	16:0/18:1	718.5409	$C_{39}H_{76}NO_8P$	804239	0.8
PE 35:2	17:0/18:1	730.5411	$C_{40}H_{76}NO_8P$	291277	0.3
PE 36:2	18:1/18:1	744.5492	$C_{41}H_{78}NO_8P$	2182317	2.3
PE 38:6	16:0/22:6	764.5172	$C_{43}H_{74}NO_8P$	1158820	1.2
PE 38:5	18:0/20:5	766.5337	$C_{43}H_{76}NO_8P$	1763964	1.9
PE 38:4	18:0/20:4	768.5506	$C_{43}H_{78}NO_8P$	1012322	1.1
PE 38:2	18:1/20:1	772.6211	$C_{43}H_{84}NO_8P$	156987	0.2
PE 39:6	17:0/22:6	778.5323	$C_{44}H_{76}NO_8P$	2609124	2.7
PE 40:7	18:1/22:6	790.5287	$C_{45}H_{76}NO_8P$	1893480	2.0
PE 40:6	18:0/22:6	792.5457	$C_{45}H_{78}NO_8P$	4423247	4.7
PE 40:5	18:0/22:5	794.5604	$C_{45}H_{80}NO_8P$	1696335	1.8
PE 40:2		800.6117	$C_{45}H_{86}NO_8P$	134525.0	0.1

Table S2. The composition of molecular species of phosphatidylcholine (PC) from the clams.

PL	Symbol	M+	Formula	Area	% in PC
PC 34:1	a17:0/17:1	746.5933	$C_{42}H_{84}NO_7P$	165641	0.1
PC 36:4		768.5833	$C_{44}H_{82}NO_7P$	4575096	2.7
PC 36:2	a14:0/22:2	772.5198	$C_{44}H_{86}NO_7P$	1241394	0.7
PC 36:1		774.6346	$C_{44}H_{88}NO_7P$	2979147	1.8
PC 37:5		780.5825	$C_{45}H_{82}NO_7P$	367053	0.2

PC 37:4		782.6174	$C_{45}H_{84}NO_7P$	1177555	0.7
PC 38:6	a16:0/22:6	792.5846	$C_{46}H_{82}NO_7P$	7956790	4.7
PC 38:5		794.5848	$C_{46}H_{84}NO_7P$	6071400	3.6
PC 38:2		800.6423	$C_{46}H_{90}NO_7P$	1861346	1.1
PC 40:7		818.6027	$C_{48}H_{84}NO_7P$	11142834	6.6
PC 40:6	a18:0/22:6	820.6153	$C_{48}H_{86}NO_7P$	14290935	8.5
PC 30:1		704.5226	$C_{38}H_{74}NO_8P$	2979567	1.8
PC 30:0		706.5400	$C_{38}H_{76}NO_8P$	9416695	5.6
PC 32:2		730.5404	$C_{40}H_{76}NO_8P$	2128977	1.3
PC 32:1		732.5525	$C_{40}H_{78}NO_8P$	17516824	10.4
PC 34:5		752.5319	$C_{42}H_{74}NO_8P$	1297666	0.8
PC 34:4		754.5408	$C_{42}H_{76}NO_8P$	5115775	3.0
PC 34:3		756.5518	$C_{42}H_{78}NO_8P$	4902910	2.9
PC 34:1	17:0/17:1	760.5813	$C_{42}H_{82}NO_8P$	17236303	10.2
PC 36:6		778.5467	$C_{44}H_{76}NO_8P$	2273993	1.3
PC 36:5	16:0/20:5	780.5542	$C_{44}H_{78}NO_8P$	5061395	3.0
PC 36:4		782.5708	$C_{44}H_{80}NO_8P$	5088689	3.0
PC 36:2	14:0/22:2	786.6157	$C_{44}H_{84}NO_8P$	3702615	2.2
PC 38:6	16:0/22:6	806.5637	$C_{46}H_{80}NO_8P$	27052247	16.0
PC 39:6		822.5940	$C_{47}H_{84}NO_8P$	3098835	1.8
PC 40:6	18:0/22:6	834.5929	$C_{48}H_{84}NO_8P$	10024963	5.9

Table S3. The composition of molecular species of phosphatidyl serine (PS) from the clams.

PL	Symbol	M-	Formula	Area	% in PS
PS 36:1	p18:0/18:1	772.5458	$C_{42}H_{80}NO_9P$	1463470	2.2
PS 38:4	p18:0/20:4	794.5213	$C_{44}H_{78}NO_9P$	907091	1.4
PS 38:4	p18:0/20:4	794.5242	$C_{44}H_{78}NO_9P$	727150	1.1
PS 38:1	p18:0/20:1	800.5756	$C_{44}H_{84}NO_9P$	17232642	26.3
PS 39:1	p18:0/21:1	814.5899	$C_{45}H_{86}NO_9P$	4344297	6.6
PS 40:6	p18:0/22:6	818.5221	$C_{46}H_{78}NO_9P$	4579900	7.0
PS 40:6	p18:0/22:6	818.5427	$C_{46}H_{78}NO_9P$	623300	1.0
PS 40:5	p18:0/22:5	820.5443	$C_{46}H_{80}NO_9P$	2894367	4.4
PS 40:4	p18:0/22:4	822.5648	$C_{46}H_{82}NO_9P$	2834565	4.3
PS 40:2	p18:0/22:2	826.5853	$C_{46}H_{86}NO_9P$	14902400	22.7
PS 40:1	p18:0/22:1	828.6057	$C_{46}H_{88}NO_9P$	2620077	4.0
PS 34:2	17:1/17:1	758.4911	$C_{40}H_{74}NO_{10}P$	988037	1.5
PS 36:1	18:0/18:1	788.5367	$C_{42}H_{80}NO_{10}P$	1202239	1.8
PS 38:1	18:0/20:1	816.5536	$C_{44}H_{84}NO_{10}P$	669399	1.0
PS 38:1	18:0/20:1	816.5643	$C_{44}H_{84}NO_{10}P$	1127650	1.7
PS 39:6	17:0/22:6	820.494	$C_{45}H_{76}NO_{10}P$	519379	0.8
PS 40:6	18:0/22:6	834.5277	$C_{46}H_{78}NO_{10}P$	5073205	7.7

PS 40:5	18:0/22:5	836.5383	$C_{46}H_{80}NO_{10}P$	2887324	4.4

Table S4. The composition of molecular species of phosphatidylinositol (PI) from the clams.

PL	Symbol	M-	Formula	Area	% in PI
PI 36:5	16:0/20:5	855.4999	$C_{45}H_{77}O_{13}P$	1439080	2.5
PI 37:5	17:0/20:5	869.5255	$C_{46}H_{79}O_{13}P$	715877	1.3
PI 38:6		881.5084	$C_{47}H_{79}O_{13}P$	483099	0.9
PI 38:5	18:0/20:5	883.5242	$C_{47}H_{81}O_{13}P$	9309764	16.4
PI 38:4	18:0/20:4	885.5440	$C_{47}H_{83}O_{13}P$	16989549	30.0
PI 39:4	20:4/19:0	899.5606	$C_{48}H_{85}O_{13}P$	799211	1.4
PI 40:6	20:1/20:5	909.5449	$C_{49}H_{83}O_{13}P$	8953275	15.8
PI 40:5	20:1/20:4	911.5593	$C_{49}H_{85}O_{13}P$	17534519	30.9
PI 40:4		913.5701	$C_{49}H_{87}O_{13}P$	490379	0.9

Table S5. The composition of molecular species of phosphatidylglycerol (PG) from the clams.

PL	Symbol	M−	Formula	Area	% in PG
PG 32:0	16:0/16:0	721.4975	$C_{38}H_{75}O_{10}P$	26424206	100

Table S6. The composition of molecular species of ceramide aminoethylphosphonate (CAEP) from the clams.

PL	Symbol	M−	Formula	Area	% in CAEP
CAEP 34:3	16:0/d18:3	639.4817	$C_{36}H_{69}N_2O_5P$	62084084	14.6
CAEP 34:2	16:2/d18:0	641.4994	$C_{36}H_{71}N_2O_5P$	74898407	17.6
CAEP 34:1	16:0/d18:1	643.5137	$C_{36}H_{73}N_2O_5P$	1094052	0.3
CAEP 34:1		643.5145	$C_{36}H_{73}N_2O_5P$	54701373	12.9
CAEP 35:3	16:0/d21:3	653.4990	$C_{37}H_{71}N_2O_5P$	40643458	9.6
CAEP 35:2	17:0/d18:2	655.5124	$C_{37}H_{73}N_2O_5P$	24780958	5.8
CAEP 35:1	16:0/d19:1	657.5291	$C_{37}H_{75}N_2O_5P$	66718626	15.7
CAEP 35:0		659.5423	$C_{37}H_{77}N_2O_5P$	6139719	1.4
CAEP 36:2		669.5300	$C_{38}H_{75}N_2O_5P$	5837542	1.4
CAEP 36:1	17:0/d19:1	671.5453	$C_{38}H_{77}N_2O_5P$	20568570	4.8
CAEP 37:3	18:0/d19:3	681.5287	$C_{39}H_{75}N_2O_5P$	36620992	8.6
CAEP 37:3	18:0/d19:3	681.5294	$C_{39}H_{75}N_2O_5P$	2260798	0.5
CAEP 37:2	18:0/d19:2	683.5487	$C_{39}H_{77}N_2O_5P$	3669098	0.9
CAEP 37:1	18:0/d19:1	685.5565	$C_{39}H_{79}N_2O_5P$	3745037	0.9
CAEP 36:3		667.4975	$C_{38}H_{73}N_2O_5P$	20841736	4.9

Supplementary B: Figures

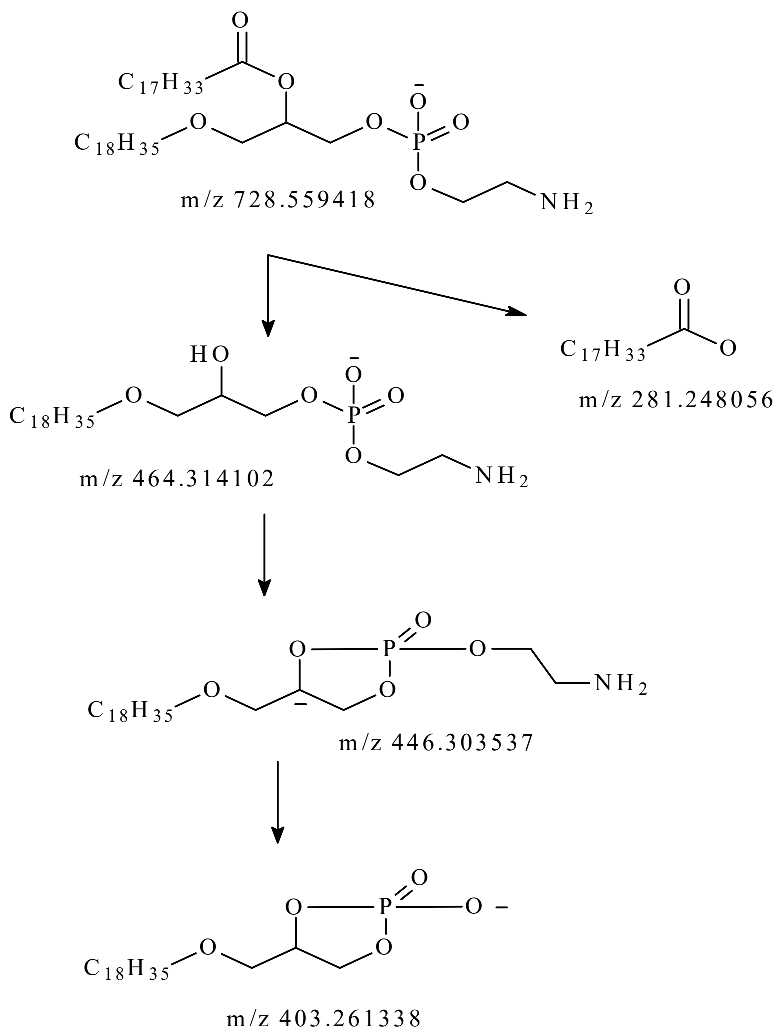

Figure S1. Fragmentation pathways of PE 36:1.

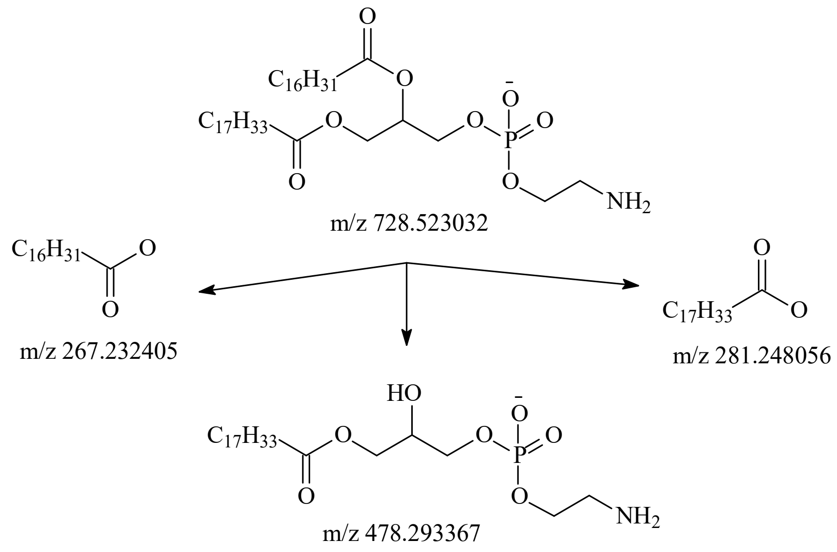

Figure S2. Fragmentation pathways of PE 35:2.

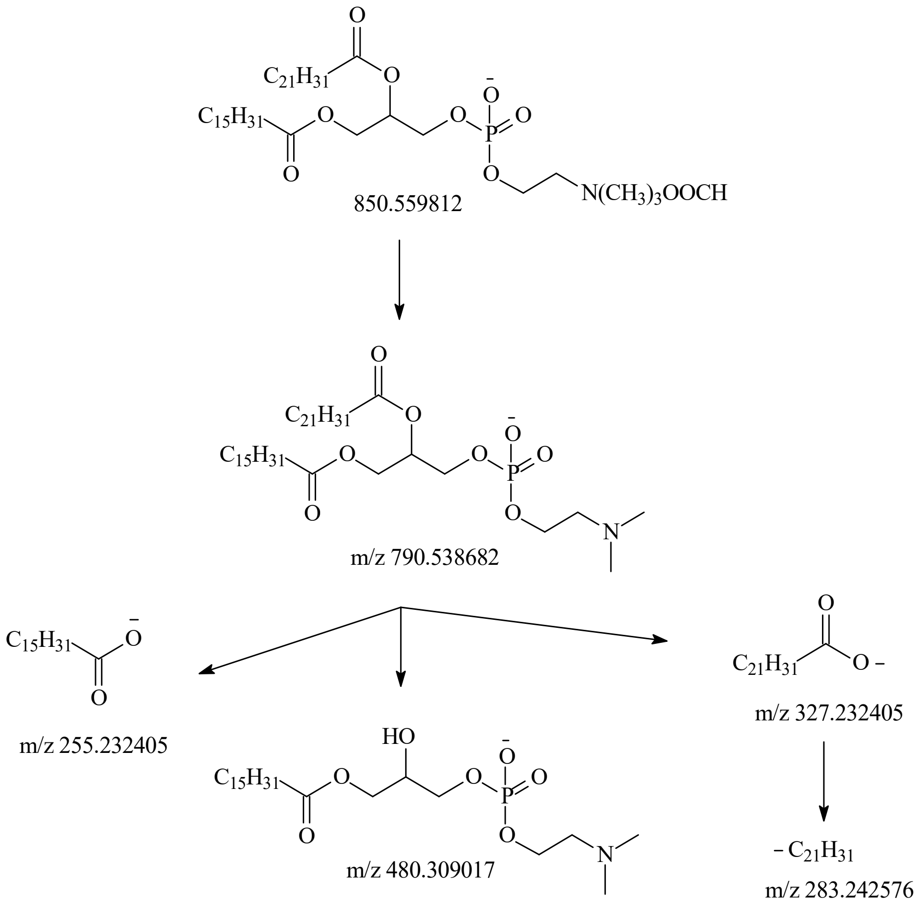

Figure S3. Fragmentation pathways of PC 38:6.

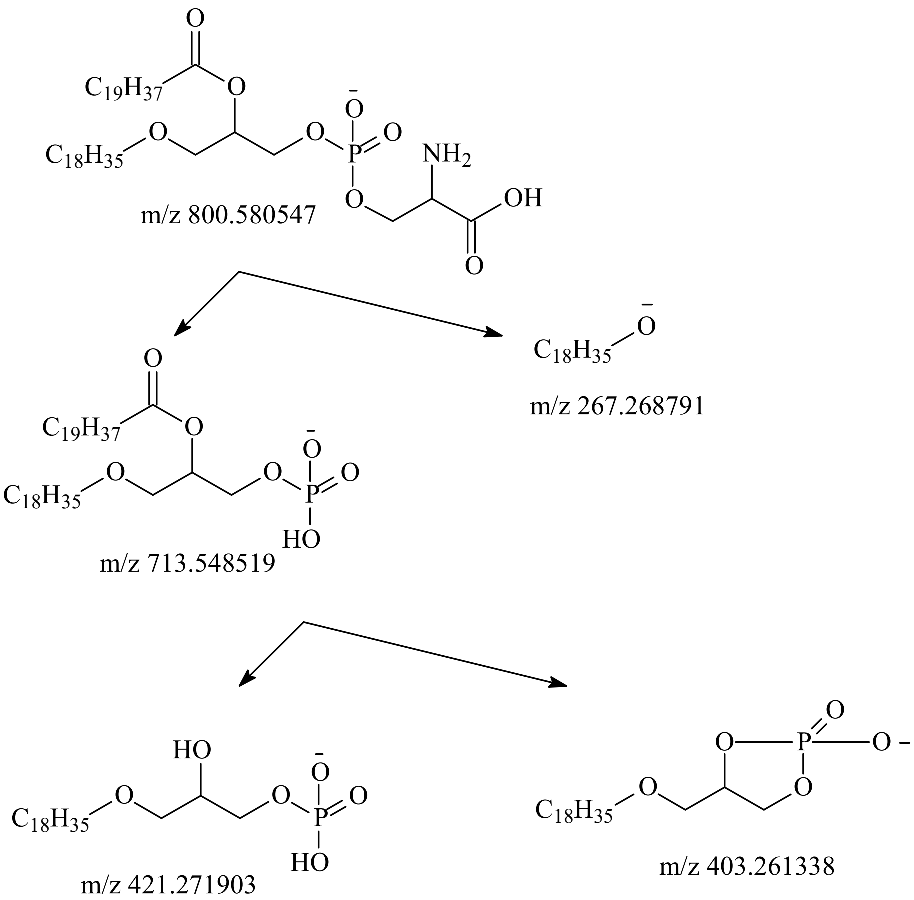

Figure S4. Fragmentation pathways of PS 38:1.

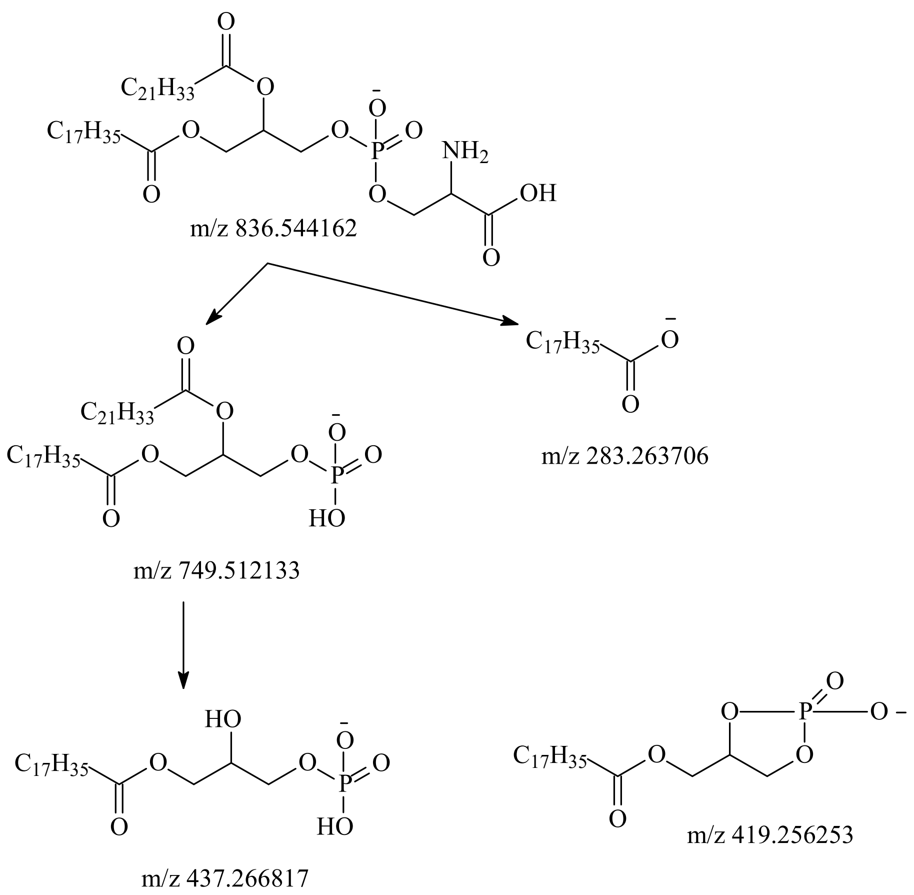

Figure S5. Fragmentation pathways of phosphotidylserine (PS 40:5).

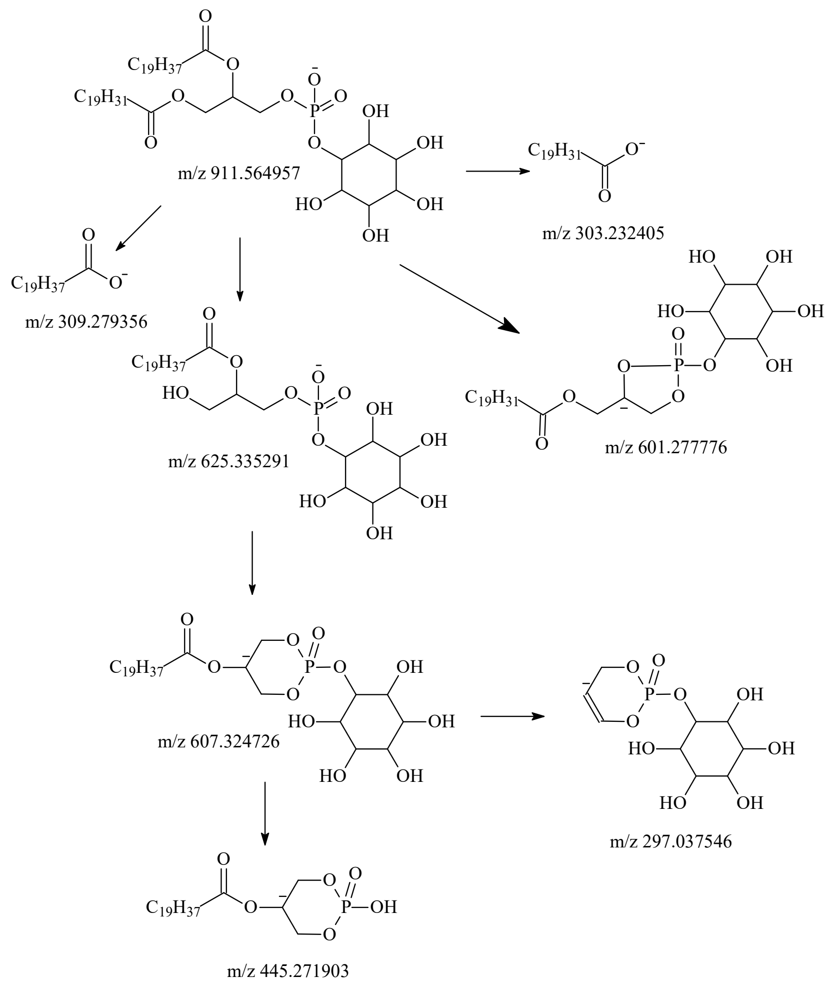

Figure S6. Fragmentation pathways of PI 40:5.

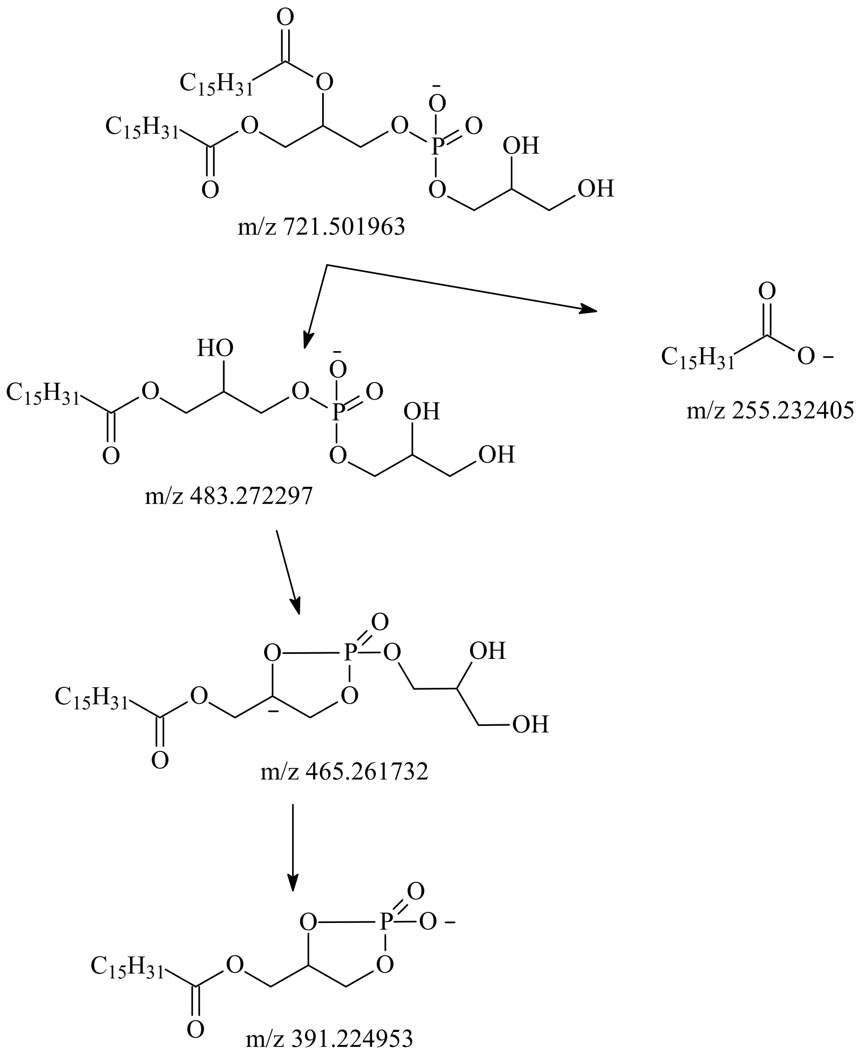

Figure S7. Fragmentation pathways of PG 32:0.

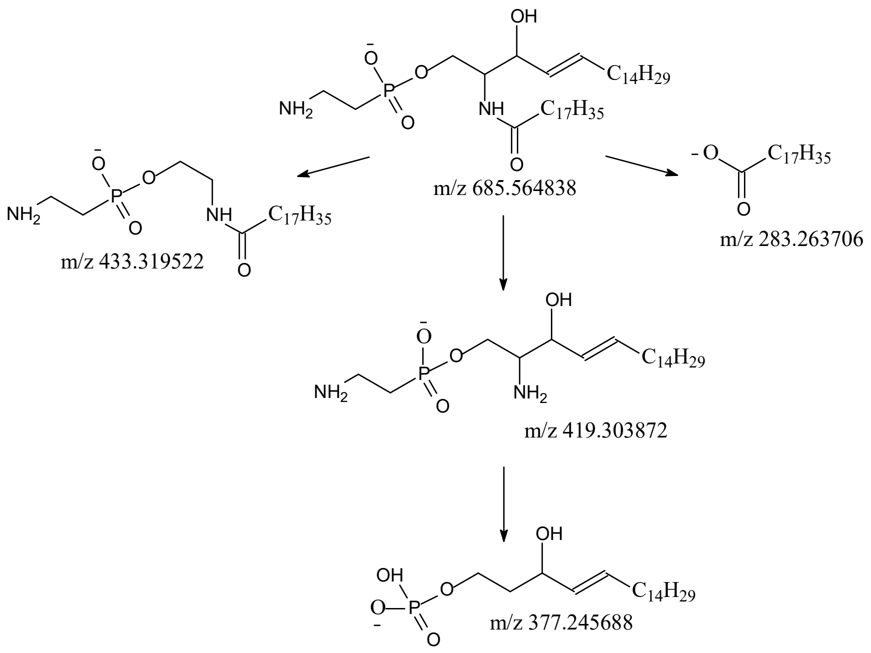

Figure S8. Fragmentation pathways of CAEP 37:1.
